# Primary Gastric Diffuse Large B-Cell Lymphoma: Prognostic Factors in the Immuno-Oncology Therapeutics Era

**DOI:** 10.4274/tjh.galenos.2020.2019.0332

**Published:** 2020-08-28

**Authors:** ZhiMin Bai, ZhenHua Li, Tao Guan, LieYang Wang, JingRong Wang, ShaoHua Wu, LiPing Su

**Affiliations:** 1 Shanxi Medical University, Taiyuan, Shanxi, China; 2 Department of Hematology, Shanxi Tumor Hospital affiliated to Shanxi Medical University, Taiyuan, Shanxi, China

**Keywords:** Primary gastric diffuse large B-cell lymphoma, CD4:CD8 ratio, Prognostic factors

## Abstract

**Objective:**

This study aimed to explore the prognostic factors for primary gastric diffuse large B-cell lymphoma (PG-DLBCL).

**Materials and Methods:**

This retrospective study analyzed 72 PG-DLBCL patients between January 2012 and December 2017 in the Shanxi Cancer Hospital of Shanxi Medical University to identify the different prognostic factors in PG-DLBCL. The clinical features, treatment, and follow-up information were analyzed.

**Results:**

The low CD4:CD8 ratio group (median subsequent overall survival [OS]: 36.06 months; 95% confidence interval [CI]: 25.73-46.40) showed a significant decrease in subsequent OS compared to the normal group among PG-DLBCL patients who were newly diagnosed and did not receive rituximab (median OS: 52.58 months; 95% CI: 44.18-60.97; p=0.029). Event-free survival status 24 months after the date of diagnosis (EFS24) also decreased significantly in the low CD4:CD8 group (median EFS24: 16.27 months; 95% CI: 13.09-19.45) compared to the normal group (median EFS24: 20.34 months; 95% CI: 17.05-23.63; p=0.014). Multivariate analysis showed that low CD4:CD8 at diagnosis was an independent poor prognostic factor for subsequent OS and EFS24.

**Conclusion:**

Our data suggest that identifying prognostic factors, especially host immunity, may provide useful information for assessing prognosis or clinical management.

## Abstract (tr)

**Amaç:**

Bu çalışmada primer gastrik diffüz büyük B hücreli lenfoma (PG-DLBCL) için prognostik faktörler araştırıldı.

**Gereç ve Yöntemler:**

Bu retrospektif çalışmada, Shanxi Tıp Üniversitesi’nin Shanxi Kanser Hastanesi’nde Ocak 2012 ile Aralık 2017 arasında 72 PG-DLBCL hastası incelendi ve PG-DLBCL’deki farklı prognostik faktörler incelendi. Klinik özellikler, tedavi ve takip bilgileri analiz edildi.

**Bulgular:**

Yeni tanı almış ve rituksimab kullanmamış PG-DLBCL hastalarında, CD4: CD8 oranı düşük olan grupta genel sağkalım (medyan genel sağkalım [OS]: 36,06 ay; %95 güven aralığı [GA]: 25,73-46,40) normal gruba kıyasla (medyan OS: 52,58 ay;% 95 GA: 44,18-60,97; p=0,029) anlamlı olarak düşük bulundu. Tanı tarihinden 24 ay sonraki olaysız sağkalım (EFS24) gene CD4: CD8 düşük olan grupta (ortanca EFS24: 16,27 ay; %95 GA: 13,09-19,45) normal gruba (ortanca EFS24: 20,34; %95 GA: 17,05-23,63; p=0,014) göre önemli ölçüde düşüktü. Çok değişkenli analiz, tanıdaki düşük CD4: CD8 oranının sonraki OS ve EFS24 için bağımsız bir kötü prognostik faktör olduğunu gösterdi.

**Sonuç:**

Verilerimiz, özellikle konakçı bağışıklığı olmak üzere prognostik faktörlerin tanımlanmasının, prognozu veya klinik yönetimi değerlendirmek için yararlı bilgiler sağlayabileceğini düşündürmektedir.

## Introduction

Primary gastric diffuse large B-cell lymphoma (PG-DLBCL) is an uncommon gastric malignancy but is the most common subtype of extranodal non-Hodgkin lymphoma (NHL), accounting for 40%-70% of all primary gastric NHL [1,2]. PG-DLBCL is a high-grade type of gastric lymphoma, more common than mucosa-associated lymphoma tissue (MALT) that entails low-grade lesions and is usually secondary to *Helicobacter pylori* infection. Patients with PG-DLBCL tend to present common symptoms including epigastric pain, nausea, vomiting, weight loss, and gastrointestinal bleeding, and all of them have a relatively favorable prognosis [3].One of the many pathogenic factors that account for PG-DLBCL is host immune deficiency. Host outcomes are typically predicted with the Lugano staging system for GI tract lymphoma and the International Prognostic Index (IPI) for aggressive NHL [4,5]. Accurate diagnoses of PG-DLBCL are generally confirmed by histopathological and immunohistochemical examination, supplemented by cytogenetic and molecular biology tests [6]. Chihora et al. [7] showed that the general prognosis of PG-DLBCL involved tumor characteristics and host-related factors, such as histological subtype, age, performance status, and host nutrition. With the advent of novel immune-based therapies, it is necessary to evaluate the prognosis of PG-DLBCL patients in terms of host immunity. Previous research has shown that achieving event-free survival status 24 months after the date of diagnosis (EFS24) predicts excellent outcome regardless of the status of established baseline prognostic factors in classic Hodgkin lymphoma [8]. Maurer et al. [9] considered EFS24 to be an endpoint for future studies of newly diagnosed DLBCL. On the basis of these findings, we used EFS24 and subsequent overall survival (OS) as our analytic primary endpoints.

The aim of this retrospective research was to identify prognostic factors, and in particular the CD4:CD8 ratio at first diagnosis, representing host immunity, that may be useful in selecting patients for clinical trials for immuno-oncology therapeutics.

## Materials and Methods

### Patients

This retrospective study included 72 patients who were diagnosed with PG-DLBCL from January 2012 to December 2017 in the Shanxi Cancer Hospital of Shanxi Medical University. The deadline for observation was 31 May 2018. The pathological specimens were obtained from endoscopic biopsies and surgical resections. The diagnosis of PG-DLBCL was established according to histopathologic and immunohistochemical criteria based on the 2017 WHO classification system. Cell-of-origin (COO) subtype was classified by the Hans criteria [10]. All the patients were staged according to the Lugano staging system for gastrointestinal NHL [4]. The clinical and laboratory data recorded for each patient, including age, sex, presenting symptoms, performance status (PS), increased lactate dehydrogenase (LDH) (>245 U/L) level, low serum albumin (<35 g/L), high β2-macroglobulin (β2-MG) (>3 mg/L), Ki67, absolute lymphocyte count (ALC) at the time of diagnosis, CD4:CD8 ratio at diagnosis, type of treatment, IPI score, and immunophenotype by the Hans algorithm, were obtained from the medical records of the institution.

### Immunophenotyping of Lymphocytes

We collected about 5 mL of venous blood of each patient at diagnosis in heparin anticoagulation vacuum blood collection tubes. The CD4^+^ and CD8^+^ T-lymphocyte numbers and CD4:CD8 ratio (1-2) were measured with the MultiTEST IMK Kit (BD, Franklin Lakes, NJ, USA) by the single platform via FACSCalibur flow cytometry (BD). In the analyzed samples, lymphocytes were defined via the gating method by size and cell granulation (forward scatter vs. side scatter). After that, lymphocyte subpopulations were determined by fluorescence on dual-parameter histograms. A total of 10,000 cells were analyzed and the results were expressed as the percentage of the examined cells in each subpopulation of lymphocytes. Cell subpopulations were analyzed with the DIVA program, version 6.1.3 (DB Bioscience, San Jose, CA, USA).

### Primary Endpoints

Event-free survival (EFS) was calculated from the date of diagnosis to the date of treatment failure, relapse, evidence of disease progression, or death due to any cause. EFS24 was defined as EFS status 24 months after the date of diagnosis. Subsequent OS from failing EFS24 was defined as the time from EFS24 failure to death or last follow-up. Subsequent OS from achieving EFS24 was defined as the time from achieving EFS24 to death or last follow-up. Previous studies have shown that EFS24 is a clinically useful endpoint in diffuse large B-cell lymphoma. Hence, we assess EFS24 and subsequent OS as primary endpoints of our analysis.

### Statistical Analysis

The Kaplan-Meier method was used for survival estimations and the log-rank test for survival comparisons. The characteristics of the two groups were compared using the chi-square test. All variables that influenced the prognosis (p<0.05) in univariate analysis were put into a multivariate analysis using the Cox regression model to determine independent prognostic factors for survival. All statistical analyses were performed using SPSS 25.0 software (IBM Corporation, Armonk, NY, USA) and statistical significance was considered at p<0.05.

## Results

### Clinical Characteristics of PG-DLBCL

Patients’ characteristics are detailed in Table 1. The group of patients included in the study consisted of 38 males and 34 females. The median age was 54 years (range: 18 to 83 years) for the whole group. The PS was good for most patients (PS 0-1 for 71%). Serum LDH and β2-MG were determined for most patients and were usually within normal limits. Among the patients evaluated endoscopically (n=72), the most commonly involved site was the corpus (38/72, 53%), followed by the antrum (31/72, 43%) and fundus (3/72, 4%). All patients were staged according to the Lugano staging system, which is a modified version of the Ann Arbor criteria for primary gastrointestinal NHL. Thirty-three patients (46%) were in Lugano stage I/II1 and 39 patients (54%) were in Lugano stage II2/IIE/IV; 20 patients (28%) had an IPI of high-intermediate/high (HI/H).

### Histopathological and Immunohistochemical Analysis

**Figure 1:**
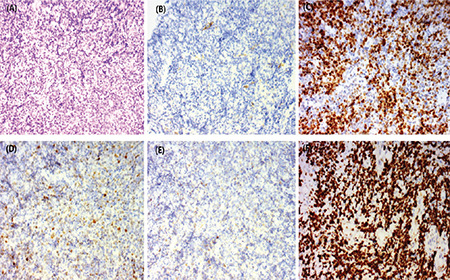
(A) Histopathology showed a large number of single lymphoid cells infiltrating the gastric mucosa (H&E staining, 200^x^). Immunohistochemical staining of primary gastric diffuse large B-cell lymphoma: (B) negative CD10 protein expression (200^x^), (C) positive Bcl-6 protein expression (200^x^), (D) about 30% positive MUM1 protein expression (200^x^), (E) about 10% positive Bcl-2 protein expression (200^x^), (F) diffuse and intense positivity of Ki67 (200^x^).

Histopathology showed diffuse infiltration of medium to large atypical lymphocytes (Figure 1). In terms of immunohistochemistry, expression of CD20 in lymphoma tissue could be detected in all patients, while only 32% (18/56) expressed CD10, 74% (34/46) Bcl-6, 65% (17/26) Bcl-2, and 66% (35/53) MUM1. Median proliferation index Ki67 was 70%, ranging from 20% to 95%. Univariate analysis of the expression of each immunophenotype biomarker and its relationship to survival showed that CD10, Bcl-6, and MUM1 were not statistically significant for subsequent OS. According to the Hans classification [10], 22 patients (30%) had germinal center B-cell (GCB) type, 35 patients (49%) had non-GCB type, and the type for 15 patients (21%) was unclear. The results of the statistical analysis of the association of immunophenotype classification and clinical characters are shown Table 2. Subclassification was not associated with Lugano stage, IPI scores, or extranodal involvement. However, a significant association existed between subclassification and CD4:CD8 ratio. Low CD4:CD8 ratio was present in 39% (7/18) of the GCB cases and 68% (23/34) of the non-GCB cases (χ^2^=3.988, p<0.05).

### Treatment Modalities and Prognostic Factors of PG-DLBCL in the Rituximab Era

Among the 72 patients, 65 patients received chemotherapy and the remaining 7 patients chose surgery only or symptomatic supportive treatment due to severe illness, poor economic condition, advanced age, and other factors. Of the 65 patients who received chemotherapy, 50 patients received chemotherapy alone and 15 patients received chemotherapy combined with surgery. Due to the patients’ poor economic situations, among the 65 PG-DLBCL patients who received chemotherapy, 25 patients were treated with rituximab (at least 4 cycles) and 40 patients were not. Among those 40 patients, CHOP-like regimens, including CHOP (cyclophosphamide, doxorubicin, vincristine, and prednisone), COP (cyclophosphamide, vincristine, prednisone), and CHOP-E (cyclophosphamide, doxorubicin, vincristine, prednisone, and etoposide), were the most frequently used first-line treatments, while other patients were treated with second-line regimens such as DHAP (dexamethasone, cisplatin, cytarabine), ICE (ifosfamide, carboplatin, etoposide), GDP (gemcitabine, dexamethasone, cisplatin), and EPOCH (etoposide, pirarubicin, vincristine, cyclophosphamide, hydroprednisone) because of disease progression or recurrence. The 5-year overall survival rate with treatments containing rituximab was 67.0% and it was 56.7% for those without rituximab (p=0.21). The 5-year progression-free survival rate was 58.3% with treatments containing rituximab and 48.7% for those without rituximab (p=0.13).

A univariate analysis identified 4 prognostic factors for survival in patients with PG-DLBCL who received CHOP or CHOP-like regimens combined with rituximab (Table 3). Based on these 4 factors, survival was unfavorable for patients with IPI scores of ≥3 (p=0.011), high β2-MG levels (p=0.003), elevated LDH levels (p=0.002), Lugano staging of ≥II2 (p=0.259), and low CD4:CD8 ratios (<1) at diagnosis (p=0.236). A multivariate analysis indicated that elevated LDH level (p=0.017) was the only independent adverse prognostic factor.

### Survival and Prognostic Factors for All PG-DLBCL Patients

**Figure 2:**
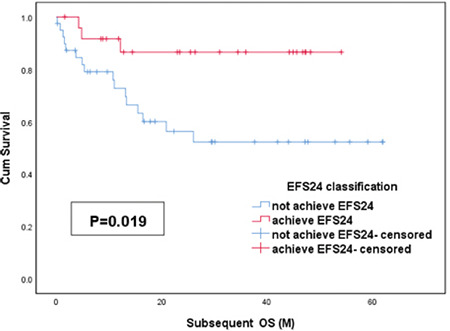
The survival difference between patients who achieved EFS24 and did not achieve EFS24 was statistically significant (p=0.019). Patients with early relapse of PG-DLBCL have poor subsequent survival.

As of 31 May 2018, twenty-three of the 72 patients (32%) had died in a median follow-up period of 33 months (range: 1-78 months). An actuarial analysis showed that the 24-month estimate of overall survival was 76.3%, with a mean survival time of 55 months (95% CI: 48-63), and 35% of patients achieved EFS24 (Kaplan-Meier estimate) with 65% failing to achieve EFS24. Patients with early relapse of PG-DLBCL had poor subsequent survival. Patients who achieved EFS24 had a mean subsequent OS of 48 months (95% CI: 42-54) versus 37 months (95% CI: 28-46) for patients who failed to achieve EFS24 (p=0.019). There was a statistically significant difference between the two groups (p<0.05) (Figure 2).

**Figure 3:**
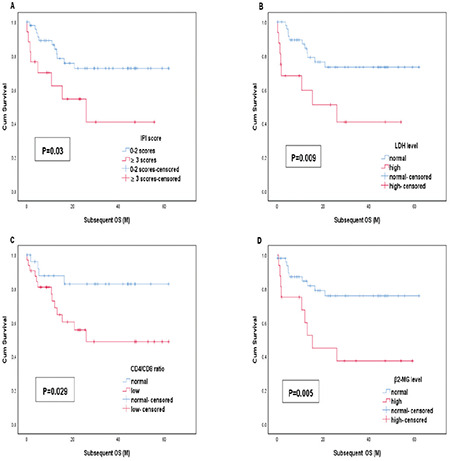
Prognostic indicators for subsequent OS in PG-DLBCL patients. Univariate analysis demonstrated that (A) IPI score of ≥3, (B) elevated LDH level, (C) low CD4:CD8 ratio at diagnosis, and (D) high β2-MG level were risk factors for PG-DLBCL. OS, Overall survival; PG-DLBCL: primary gastric diffuse large B-cell lymphoma; IPI: International Prognostic index; LDH: lactate dehydrogenase.

In univariate analysis, elevated LDH level, IPI of ≥3, high β2-MG level, and low CD4:CD8 ratio (<1) at diagnosis were related to subsequent overall survival (Figure 3). In multivariate analysis of subsequent OS, low CD4:CD8 ratio (relative risk [RR]: 3.26, 95% CI: 1.06-10.06, p=0.039) remained a significant predictor in PG-DLBCL patients who were newly diagnosed and did not receive rituximab. Table 4 summarizes univariate and multivariate analyses of the factors considered as predictors of subsequent OS.

**Figure 4:**
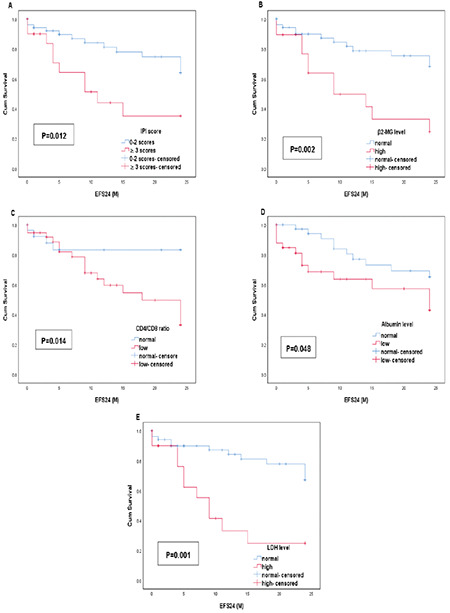
Univariate analysis demonstrated that (A) IPI score, (B) β2-MG level, (C) CD4:CD8 ratio, (D) albumin level, and (E) elevated LDH level were predictive variables of EFS24 in PG-DLBCL patients. IPI: International Prognostic Index, LDH: lactate dehydrogenase, PG-DLBCL: primary gastric diffuse large B-cell lymphoma.

Possible predictive variables of EFS24 were also estimated by univariate and multivariate analyses as shown in Table 5. We found that IPI score, β2-MG level, CD4:CD8 ratio, albumin level, and elevated LDH level were statistically significant for EFS24 (Figure 4). Elevated LDH level (RR: 3.03, 95% CI: 1.25-7.34, p=0.014) and low CD4:CD8 ratio at diagnosis (RR: 3.17, 95% CI: 1.04-9.66, p=0.042) were effective predictors of EFS24 in Cox multivariate analysis.

## Discussion

This study aimed to clarify the significant prognostic factors associated with PG-DLBCL and define the characteristics of patients with a poor prognosis to facilitate patient selection for clinical trials in the current era of immuno-oncology. In this study, there were 72 patients with PG-DLBCL, 53% of whom were male and 47% female. This male predominance is in accordance with previous observations [11]. Our research found that PG-DLBCL patients with early relapse have poor subsequent OS. Patients who achieved EFS24 had a more favorable subsequent OS than patients who failed to achieve EFS24. Therefore, assessment of EFS24 may stratify subsequent outcomes in PG-DLBCL patients. Reassessment of patient status at 24 months from diagnosis is a strong predictor of subsequent OS in PG-DLBCL patients.

Previous studies have described several features that served as prognostic indicators of poor clinical outcome in PG-DLBCL, including deficiency of gene translocation involving the immunoglobulin heavy chain [12], Helicobacter pylori negativity [13], Lugano stage II2/IIE/IV, elevated serum LDH level [14], and Epstein-Barr virus infection [15]. Due to the marked heterogeneity of DLBCL, a reliable prediction tool is vital for optimizing patient treatment. In our study, elevated LDH level, IPI score of ≥3, high β2-MG level, and low CD4:CD8 ratio at diagnosis were associated with EFS24 and subsequent OS, while low CD4:CD8 ratio at diagnosis was an independent prognostic indicator for both EFS24 and subsequent OS. Since very few studies have analyzed the significance of the CD4:CD8 ratio at the time of diagnosis in PG-DLBCL, it is necessary to explore the predictive value of low CD4:CD8 ratios in PG-DLBCL. The CD4:CD8 ratio has been used as a surrogate marker of immunosenescence in the general population [16,17]. In addition, the CD4:CD8 ratio naturally decreased with age, was associated with increased mortality, and was a marker of both acute and chronic inflammation [18,19]. The CD4:CD8 ratio, representing host immunity, may play a role in preventing the progression of PG-DLBCL. We found that the CD4:CD8 ratio at diagnosis was prognostic for both EFS24 and subsequent OS, because patients with low CD4:CD8 ratios at the time of diagnosis had a poorer clinical outcome and shorter survival than those with higher ratios. The link between a persistently abnormal CD4:CD8 ratio and worse outcomes in PG-DLBCL patients is complex. CD4^+^ thymus-derived naturally occurring regulatory T-cells (Treg cells) play an indispensable role for the maintenance of self-tolerance and immune homeostasis, and Treg cells stimulated by transforming growth factor-beta (TGF-β) differentiated from original CD4^+^ T-cells and participated in immune regulation [20]. García et al. suggested that Treg cells have stronger impact in treatments that are not directly targeted against the tumor cells [21]. This impact is probably due to the role of Tregs in modulating tumor growth through different mechanisms such as suppressing the activity of antigen-presenting T-cells. Therefore, a low CD4:CD8 ratio may reduce the number of Treg cells, which decreases the inhibitory effect on the tumor. We recommend that immune function in patients with PG-DLBCL be routinely examined to provide better assessments of prognosis and better predictions of the therapeutic effects of immuno-oncology.

Almost all nucleated cell membranes express a kind of human leukocyte antigen-class I molecule called β2-MG [22]. Huang et al. [23] reported that β2-MG is a growth factor and signaling molecule in cancer cells by regulating p21-activated kinases, VEGF, and fatty acid synthase-mediated growth and survival signaling pathways [24]; it also plays an important role in the proliferation, apoptosis, and metastasis of cancer cells [23,25]. Furthermore, β2-MG is a useful prognostic factor in multiple types of cancer, including diffuse large B-cell lymphoma [26], carcinoma of the breast [27], chronic lymphocytic leukemia [28], and carcinoma of the gallbladder [29]. In this study, our results were in agreement with previous research that PG-DLBCL patients with higher β2-MG levels have poorer prognoses than those patients with normal levels. As for LDH level, our study was in accordance with previous research finding that elevated LDH levels always implied poor prognosis [11]. In addition, several studies reported that serum albumin level is a prognostic factor in patients with DLBCL [30,31,32], while in our study, serum albumin levels (<35 g/L), which more likely represented disease severity, were associated with patients failing to achieve EFS24 but not with poor subsequent OS.

Despite the fact that rituximab improves survival rates and clinical responses in patients with nodal DLBCL when used with conventional chemotherapy [33,34], in our study, there was no statistically significant difference between the patients with and without rituximab treatment, which may be due to the limited use of rituximab and the small number of specimens. Moreover, since the R-CHOP chemotherapy regime was the standard therapy for DLBCL, we conducted a separate analysis of patients receiving R-CHOP to confirm the effects of these factors, including IPI scores of ≥3, high β2-MG level, elevated LDH level, and low CD4:CD8 ratio (<1) at diagnosis on prognosis. Except for the low CD4:CD8 ratio, the remaining three prognostic factors were consistent with the results of the overall study. This may be due to the regulatory effect of rituximab on immune function. Many studies have found that rituximab has an effect on lymphocytes and the CD4:CD8 ratio. For example, Zhang et al. [35] found that low CD4:CD8 ratio was associated with poor OS in patients with mantle cell lymphoma. In addition, in cases of granulomatosis with polyangiitis, the CD4:CD8 ratio decreased slightly after initial rituximab treatment and gradually increased during rituximab maintenance [36]. In our study, low CD4:CD8 ratio was associated with poor OS in PG-DLBCL patients who were newly diagnosed and did not receive rituximab, but in patients receiving rituximab, low CD4:CD8 ratio was not associated with poor OS. Such a result may be related to the effect of rituximab on lymphocytes. Therefore, in PG-DLBCL without rituximab, low CD4:CD8 ratio was associated with poor prognosis, while in patients with rituximab maintenance therapy, the CD4:CD8 ratio could not be identified as a prognostic factor.

A recent study has proven that immunophenotype is an important independent prognostic factor [37]. We used the Hans algorithm to classify the PG-DLBCL patients in our study. We examined the relationships among immunophenotype classification with Lugano stage, IPI scores, performance status, and low CD4:CD8 ratio. We found that non-GCB patients were more likely to have low CD4:CD8 ratios than GCB patients. However, there was no correlation between immunophenotype classification and survival time, which was in accordance with the study by Dwivedi et al. [38].

## Conclusion

In conclusion, we identified the following factors with prognostic impact for the studied group: low CD4:CD8 ratio at diagnosis was associated with both the status of EFS24 and poor subsequent overall survival, while high LDH level was an effective predictor of EFS24 status. Our data suggest that EFS24 status provides a simple, clinically relevant endpoint for studies assessing outcome in PG-DLBCL patients. Moreover, the CD4:CD8 ratio, which represents the host’s immunity, was superior compared to other prognostic factors and may be useful in selecting patients for clinical immuno-oncology therapeutics.

## Figures and Tables

**Table 1:**
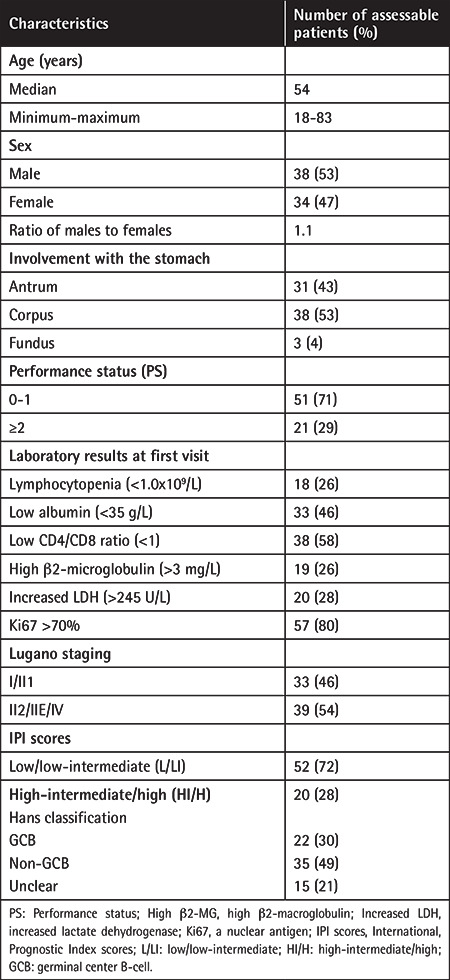
Baseline characteristics of 72 patients with PG-DLBCL.

**Table 2:**
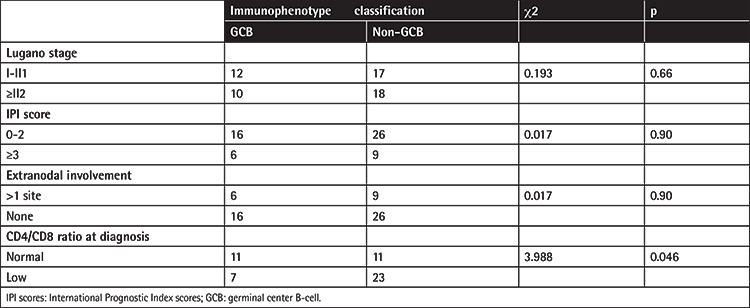
Correlation between immunophenotype classification and clinical characters.

**Table 3:**
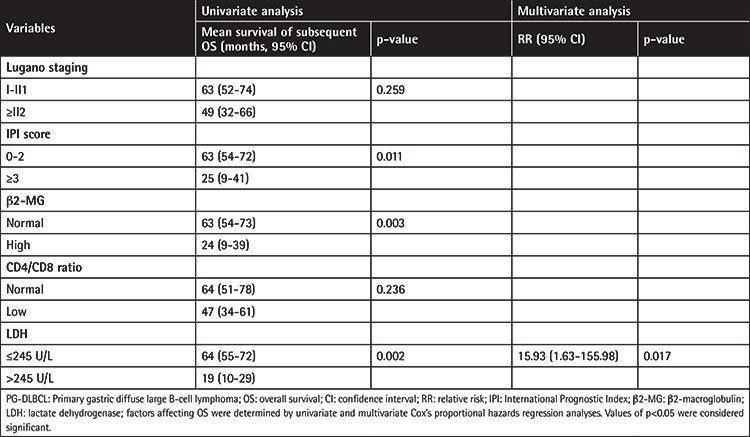
Univariate and multivariate analysis for OS in PG-DLBCL patients with rituximab-containing chemotherapy.

**Table 4:**
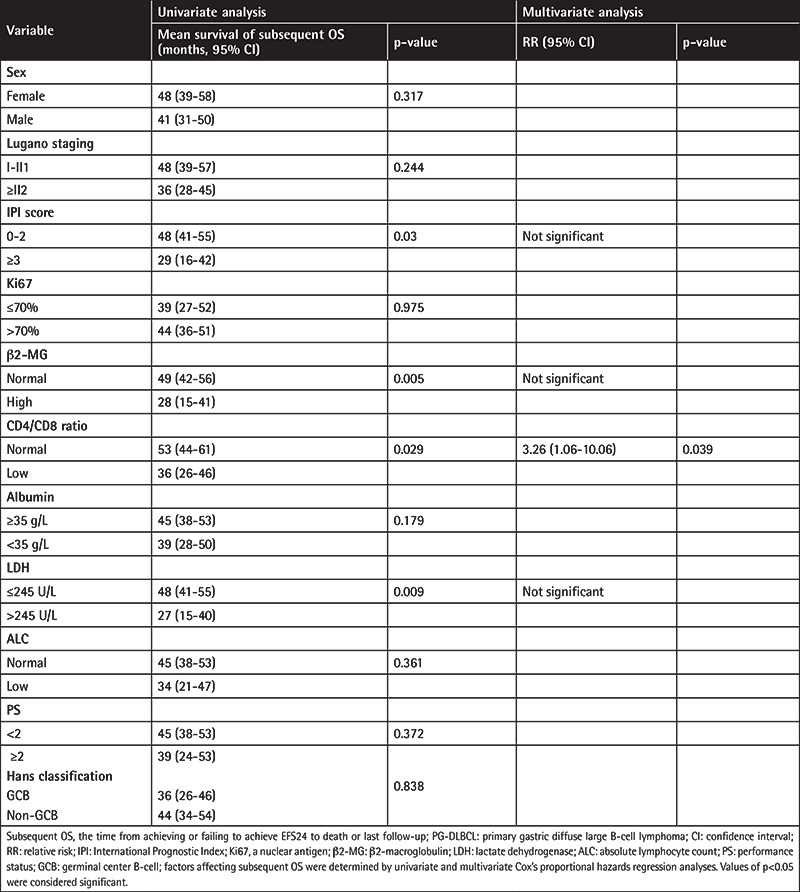
Risk factors associated with subsequent OS in PG-DLBCL patients.

**Table 5:**
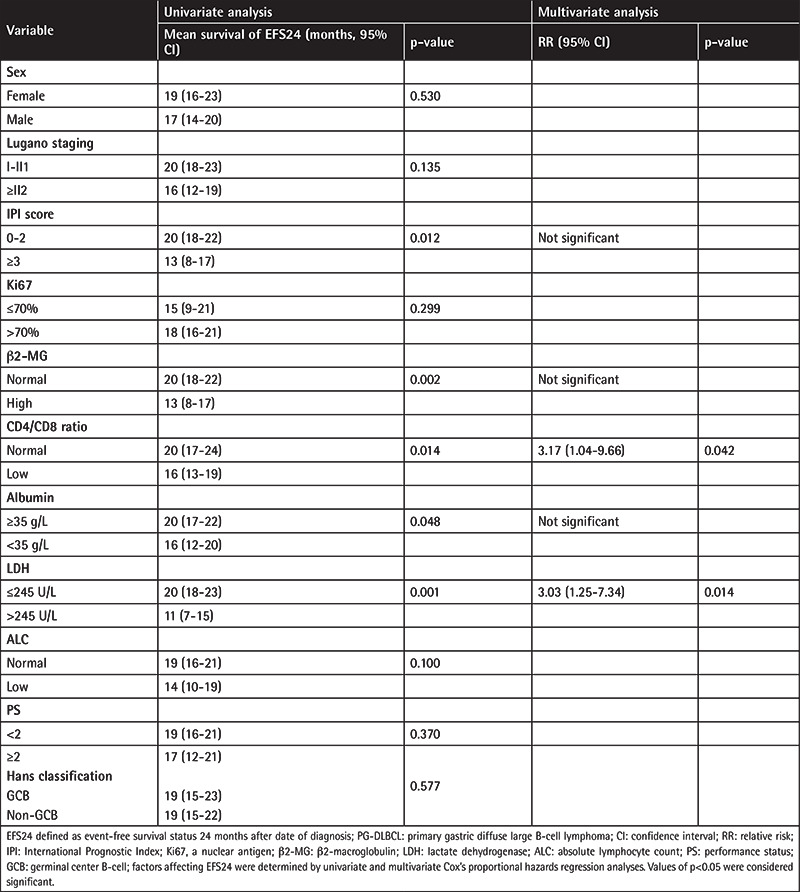
Risk factors associated with EFS24 in PG-DLBCL patients.
